# Valorization of *Sicana* *odorifera* (Vell.) Naudin Epicarp as a Source of Bioactive Compounds: Chemical Characterization and Evaluation of Its Bioactive Properties

**DOI:** 10.3390/foods10040700

**Published:** 2021-03-25

**Authors:** Bianca R. Albuquerque, Maria Inês Dias, Carla Pereira, Jovana Petrović, Marina Soković, Ricardo C. Calhelha, M. Beatriz P. P. Oliveira, Isabel C. F. R. Ferreira, Lillian Barros

**Affiliations:** 1 Centro de Investigação de Montanha (CIMO), Instituto Politécnico de Bragança, Campus de Santa Apolónia, 5300-253 Bragança, Portugal; bianca.albuquerque@ipb.pt (B.R.A.); maria.ines@ipb.pt (M.I.D.); carlap@ipb.pt (C.P.); calhelha@ipb.pt (R.C.C.); iferreira@ipb.pt (I.C.F.R.F.); 2 REQUIMTE—Science Chemical Department, Faculty of Pharmacy, University of Porto, Rua Jorge Viterbo Ferreira 228, 4050-313 Porto, Portugal; beatoliv@ff.up.pt; 3 Institute for Biological Research “Siniša Stanković”, Department of Plant Physiology, University of Belgrade, Bulevar Despota Stefana 142, 11000 Belgrade, Serbia; jovana0303@ibiss.bg.ac.rs (J.P.); mris@ibiss.bg.ac.rs (M.S.)

**Keywords:** fruit by-products, non-anthocyanin polyphenols, anthocyanin polyphenols, natural-based colorants, antimicrobial and antioxidant activities

## Abstract

Fruit bio-residues can be interesting for the recovery of bioactive molecules, such as phenolic compounds, tocopherols, vitamins, among others. These compounds can be targeted at the food industry and used for the development of functional foods or as food additives. In some cases, fruit epicarps are converted into by-products with non-commercial value, and generally, these fruit parts have a higher content in bioactive compounds than the fruit pulp. From this perspective, *S. odorifera*, a Brazilian fruit, has an inedible epicarp that could be explored to obtain biological compounds. Therefore, the aims of this study were to evaluate the chemical composition and the antioxidant, anti-proliferative, anti-inflammatory, and antimicrobial bioactivities of this by-product. *S. odorifera* epicarp showed a total of four organic acids, four phenolic compounds, highlighting the high concentration of anthocyanins (24 ± 1 mg/g dry weight (dw)) and high content of tocopherols (366 ± 2 mg/100 g dw). The hydroethanolic extract showed considerable antioxidant activity (EC_50_ values of 48.2 ± 0.5 and 27 ± 1 µg/mL for TBARS and OxHLIA assays, respectively), as also antibacterial and antifungal activities (minimal inhibitory concentrations (MICs) ≤ 2.2 mg/mL). The results obtained in this study suggest that *Sicana odorifera* epicarp represents a reliable option for the development of novel natural-based colorants with functional/bioactive proprieties.

## 1. Introduction

Bio-residues from fruits and vegetables generated during post-harvest treatments and processing steps, can be considered as interesting matrices for the recovery of bioactive molecules, such as phenolic compounds, organic acids, tocopherols, vitamins, dietary fibers, and fatty acids [1,2]. All of these molecules, with high added value in the market, can be potentially used in several industrial sectors, such as in the food processing industry for the formulation of novel functional foods or to be used as additives and also in the pharmaceutical industry as natural therapeutic alternatives, among others [1,2].

In the specific case of fruit processing, the peels and seeds are converted into by-products with non-commercial value, however, in most cases, these by-products contain higher amounts of phenolic compounds than the fruit pulp [3]. In fact, by-products in red-purple-colored fruits have been described as having higher amounts of anthocyanin compounds, which validates the hypothesis of its exploitation for the recovery of these molecules, for later application as coloring agent [4].

*Sicana odorifera* (Vell.) Naudin is an indigenous plant belonging to the Cucurbitaceae family, probably native from Brazil, but also widely spread in other tropical countries in Central America and South America, such as Mexico, Colombia, Peru, and Paraguay, where it is popularly known as melon croá, cajuá, cassabanana, sikana, olor melon, and jamelão [5,6,7,8]. This fruit presents a long cylindrical size (30–60 cm long and 7–11 cm in diameter), with a weight rounding between 1.4 and 3.0 kg [6,7,8,9,10]. Its fruit pulp of yellow-orange color is appreciated in not only the immature stage, cooked to accompany meals, but also in a fully ripe mature stage, consumed in-natura form or used in the preparation of jams and juices [5,8,10]. The fruit peel (epicarp) is a non-edible hard thin layer, with an orange-red, brown or dark-purple coloration [11]. Its most traditional form of use is as an insect repellent [12] and also as medicine for the treatment of liver diseases, sore throat, fever, uterine hemorrhages, and venereal diseases [10,11,13].

Although this fruit is very common in several regions of America, the scientific community does not have the most in-depth knowledge about it and all its potential. In the literature, there are a few studies about this exotic fruit. Due its pleasant unique odor, *S. odorifera* is popularly used to perfume clothes and houses [5,11], mainly due to its rich profile in volatile compounds, such as methyl-2-butanol, 3-hydroxy-2-butanone, 4-hydroxybenzyl methyl ether, and 2-phenylethanol, among others [5]. There are also a few studies regarding its nutritional value [6,7,10], and even *S. odorifera* pulp polysaccharide composition [8]. The literature regarding the non-edible (peels and seeds) parts of *S. odorifera* is even scarcer. Jaramillo et al. [8] described the flavonoids composition in different methanolic extracts fraction from epicarp; while Nakano et al. [12] isolated a new triterpene, named cucurbita-5,23-diene-3β,25-diol, from *S. odorifera* seeds.

Facing the enormous potentiality of this plant fruit bio-residues as a source of high added value compounds and in the way aim of promoting its valorization, the present work outlined as the main objective the characterization of bioactive molecules present in the epicarp of *S. odorifera*, such as organic acids, tocopherols, and phenolic compounds (non-anthocyanin and anthocyanin), by HPLC coupled to a DAD, fluorescence, and DAD-ESI/MS detectors, respectively. Furthermore, in vitro evaluation assays of its bioactive properties were performed, namely, antioxidant, anti-proliferative, antibacterial, and antifungal activities.

## 2. Materials and Methods

### 2.1. Preparation of Sample

Purple fruits of *S. odorifera* were acquired in CEAGESP—Companhia de Entrepostos e Armazéns Gerais de São Paulo (São Paulo, Brazil) from Luma Comércio e distribuidora de frutas LTDA—sociedade limitada—company (São Paulo, Brazil), marketed with the common name jamelão. The fruits were selected and washed, and the epicarps were separated from the pulp with the aid of knives. After that, the epicarps were frozen at −18 °C and further lyophilized (FreeZone 4.5, Labconco, Kansas City, MO, USA) until complete dryness (dry matter yield of 29 ± 2% in relation to the fresh weight of the sample). Subsequently, the dry epicarps were reduced to a fine and homogeneous powder using a domestic electric blender and stored at −18 °C under protection from light until further analysis.

### 2.2. Chemical Characterization

#### 2.2.1. Determination of Organic Acids

Organic acids analyses were performed accordingly to the methodology described by Barros et al. [14], using an ultra-fast liquid chromatography linked to a photodiode-array detector (UFLC-PDA, Shimadzu Corporation, Tokyo, Japan), operating under conditions previously defined [14]. Standards of oxalic acid (calibration curve (CC): *y* = 1E + 7*x* + 231,891; *R*^2^ = 0.9999, limit of detection (LOD) = 12.55 µg/mL; limit of quantification (LOQ) = 41.82 µg/mL); shikimic acid (CC: *y* = 5E + 7*x* + 109,778; *R*^2^ = 0.9999, LOD = 10.2 μg/mL; LOQ = 56.5 μg/mL); citric acid (CC: *y* = 1E + 6*x* + 10,277; *R*^2^ = 0.9997, LOD = 0.11 μg/mL; LOQ = 0.34 μg/mL); fumaric acid (CC: *y* = 1E + 8*x* + 614,399; *R*^2^ = 0.9986, LOD = 0.08 μg/mL; LOQ = 0.26 μg/mL), bought from Sigma (St. Louis, MO, USA), were used for identification and quantification of the compounds. The LabSolutions Multi LC-PDA version 1.25 software (Shimadzu Corporation, Tokio, Japan) was used for analysis the data, and the results were expressed in g/100 g of dry weight (dw).

#### 2.2.2. Determination of Tocopherols

The extraction and analysis of tocopherols present in the *S. odorifera* epicarp were performed according to a protocol established by Barros et al. [14]. For the chromatographic analysis of the compounds, a high-performance liquid chromatography system (Knauer HPLC, Smartline system 1000, Berlin, Germany), coupled to a fluorescence detector (FP-2020 Jasco, Easton, MD, USA), operating in the parameters determined by the authors, was used. Tocol standard, from Matreya LLC (Pleasant Gap, PA, USA), was used as internal standard, while authentic standards of tocopherol isoforms (α-, β-, γ-, and δ-), acquired from Sigma (St. Louis, MO, USA), were used for identification and quantification of the compounds. The results were analyzed using the Clarity 2.4 software (DataApex, Prague, Czech Republic) and were expressed in mg/100 g of dw.

#### 2.2.3. Determination of Phenolic Compounds

- Non-anthocyanin phenolic compounds extraction and HPLC-DAD-MS^n^ analysis

Lyophilized sample (3 g) was magnetic stirred with ethanol–water solution (90 mL, 80:20 *v*/*v*, 150 rpm) for 1 h at room temperature, and subsequently filtered through Whatman No. 4 paper. The residue was then re-extracted with the same solution and according to the conditions described above. The total extract obtained was filtrated through a filter paper and evaporated under reduced pressure at 40 °C in a rotary evaporator and the residual water was subsequently lyophilized.

The dry extract was re-dissolved in ethanol:water solvent (20:80 *v*/*v*) at concentration of 5 mg/mL and filtered through a 0.22 µm disposable filter disk into an amber vial for HPLC analysis. The detection of the compounds was made by liquid chromatography with diode-array detector (Dionex Ultimate 3000 HPLC, Thermo Scientific, San Jose, CA, USA) conjugated to electrospray mass ionization (HPLC-DAD-ESI/MS^n^), operating under the conditions thoroughly described by Bessada et al. [15]. Data were collected and analyzed using the Xcalibur^®^2.2. SP1.48 program (Thermo Finnigan, San Jose, CA, USA). The identification of the compounds was made based on the chromatographic data obtained and according to the scientific literature. For quantification, a standard of quercetin-3-*O*-glucoside (CC: *y* = 34,843*x* − 160,173, *R*^2^ = 0.9998, limit of detection (LOD) = 0.21 µg/mL, and limit of quantitation (LOQ) = 0.71 µg/mL) was acquired from Extrasynthèse (Genay, France). The results were expressed as mg/g of extract (E) and as mg/g of dry epicarp (dw).

- Anthocyanin compounds extraction and HPLC-DAD-MS^n^ analysis

For extraction of anthocyanin compounds, the same method previously described above was performed, however– the ethanol-water solution was acidified with 0.1% citric acid (1 µM) in order to maintain the integrity of the structure of anthocyanins.

The lyophilized extract was re-dissolved and filtrated in the same conditions described above for non-anthocyanin compounds. The analysis was made using the same HPLC-DAD-MS^n^ system described above, operating in the conditions determined by Gonçalves et al. [16]. Data were collected and analyzed using the Xcalibur^®^ program.

Anthocyanin identification were made based on chromatographic characteristics of the detected compounds and according to the literature data. For quantification, standards of cyanidin-3-*O*-glucoside (CC: *y* = 134,578*x* − 3,000,000, *R*^2^ = 0.9986, LOD = 0.10 µg/mL, and LOQ = 0.30 µg/mL) and perlargonidin-3-*O*-glucoside (CC: *y* = 61,493*x* − 628,875, *R*^2^ = 0.9957, LOD = 0.24 µg/mL and LOQ = 0.75 µg/mL), both acquired from Extrasynthèse (Genay, France), were used. Results were expressed as mg/g of extract (E) and mg/g dw.

### 2.3. Evaluation of Bioactive Properties of S. odorifera Epicarp Extract

#### 2.3.1. Antioxidant Activity

The extract obtained from *S. odorifera* epicarp was evaluated regarding its antioxidant capability using two cell-based methods. The anti-lipid peroxidation activity was assessed by the thiobarbituric acid reactive substances assay (TBARS), carried out according to the methodology described by Barros et al. [14]. As result, the concentration that provides 50% inhibition of lipid peroxidation (EC_50_ value, µg/mL) was defined. The determination of the anti-hemolytic propriety of the sample was performed by the oxidative hemolysis inhibition assay (OxHLIA), following a protocol previously described by Lockowandt et al. [17]. This methodology made it possible to determine the extraction concentration required to protects 50% of the erythrocytes, obtained from sheep blood, of the oxidative hemolysis at delay of 60 min (EC_50_ value (μg/mL) at Δt_60_ min). All analyses were performed in triplicate.

#### 2.3.2. Anti-Inflammatory Activity

The method of LPS-induced nitric oxide (NO) production by mouse macrophages RAW 264.7, previously described by Corrêa et al. [18], was applied to evaluate the anti-inflammatory activity of the extract obtained from *S. odorifera* epicarp. Dexamethasone (50 μM) was used as positive control. The concentrations needed to inhibit 50% of the NO production (EC_50_ values ((μg/mL)) were determined to express the results.

#### 2.3.3. Anti-Proliferative Activity and Hepatotoxicity

The anti-proliferative proprieties of the hydroethanolic extract were tested by Sulforodamine B (SRB) assay using four human tumor cell lines (MCF-7, breast adenocarcinoma; NCI-H460, non-small cell lung cancer; HeLa, cervical carcinoma; and HepG2, hepatocellular carcinoma), acquired from Leibniz-Institut DSMZ. As for hepatotoxicity evaluation, the extract was tested in a normal porcine liver cells (PLP2) obtained from local abattoir. The analysis were performed in triplicate, and the extract concentrations able to inhibit 50% of cell growth (GI_50_ values (μg/mL)) were determined following the protocol established by Corrêa et al. [18].

#### 2.3.4. Antibacterial Activity

Inhibitory activity of hydroethanolic extract prepared from *S. odorifera* epicarp was tested towards three Gram-positive bacterial strains: *Staphylococcus aureus* (ATCC 6538), *Bacillus cereus* (food isolate), and *Listeria monocytogenes* (NCTC 7973), as well as three Gram-negative strains: *Escherichia coli* (ATCC 35210), *Salmonella typhimurium* (ATCC 13311), and *Enterobacter cloacae* (ATCC 35030). All the tested microorganisms were obtained from the Mycological laboratory, Department of Plant Physiology, Institute for Biological research “Siniša Stanković”, University of Belgrade, National Institute of Republic of Serbia. The minimum inhibitory and bactericidal concentrations (MICs and MBCs, respectively) were obtained using microdilution assay in 96-well microtiter plates according to Soković et al. [19]. The MIC values, which indicate significant lack of bacterial growth in presence of the tested sample, were determined using colorimetric viability assay based on reduction in an INT color (p-iodonitrotetrazolium violet (Sigma, St. Louis, MO, USA). MBC values were obtained after reinoculation of the content of the well (containing medium, extract, and bacterial inoculum, 10 µL) which showed no bacterial growth into the fresh medium, and further incubation at 37 °C for 24 h. The lowest concentrations that showed no bacterial growth were defined as the MBCs, indicating 99.5% eradication of the tested bacteria. As positive controls, two commercial food preservatives were used: sodium benzoate—E211 and potassium metabisulfite—E224.

#### 2.3.5. Antifungal Activity

Regarding antifungal activity, hydroethanolic extract of *S. odorifera* epicarp was evaluated using following micromycetes: *Aspergillus fumigatus* (ATCC 1022), *Aspergillus versicolor* (ATCC 11730), *Aspergillus niger* (ATCC 6275), *Penicillium funiculosum* (ATCC 36839), *Penicillium verrucosum* var. *cyclopium* (food isolate), and *Trichoderma viride* (IAM 5061). Tested organisms were obtained from the Mycological Laboratory, Department of Plant Physiology, Institute for Biological Research “Siniša Stanković”, University of Belgrade, National Institute of Republic of Serbia. The assay was performed as previously described by Soković and Van Griensven [20]. The fungal spores used for the assay were washed from the surface of agar plates containing tested fungal strains with sterile 0.85% saline containing 0.1% Tween 80 (*v*/*v*). Subsequently, their concentration was adjusted with sterile saline to a concentration of approximately 1.0 × 10^5^ in a final volume of 100 µL per well. Prior to the experiment, fungal inocula were cultured on malt agar plates at 25 °C for 72 h to check their validity and lack of contamination. Antifungal assay was performed as follows: tested extract was poured into fresh malt extract broth, after which the appropriate concentrations of fungal inocula were added. The lowest concentrations without visible fungal growth (at the binocular microscope) were defined as MICs. The fungicidal concentrations (MFCs) were determined by reinoculation of 10 µL of medium with inoculum and tested extracts into microtiter plates containing 100 µL of fresh broth per well and further incubation for 72 h at 25 °C. The lowest concentration with no visible fungal growth was defined as MFC—a value that indicates that the tested sample inhibited 99.5% of the original fungal inoculum. The commercial food preservative E211 and E224 were used as positive controls.

## 3. Results and Discussion

### 3.1. Chemical Composition

#### 3.1.1. Organic Acids

*S. odorifera* epicarp was evaluated regarding its composition in organic acids. The results found are presented in Table 1. In total, four organic acids were detected, which sums up about 3.38 g of organic acids/100 g dw, being citric acid the most plentiful, followed by oxalic acid. Only trace amounts of shikimic and fumaric acids were found (below the limits of quantification of the calibration curves). According to the literature, ascorbic acid has been quantified in *S. odorifera* pulp (1.87 ± 0.08 mg/100 g fw—fresh weight) and in the pulp of another fruit of the same species, namely, in *Sicana sphaerica* Vell (3.21 ± 0.29 mg/100 g fw) [6]. However, in the present study, this organic acid was not detected. Data regarding the organic acid composition of the epicarp of *Sicana* spp. have not been found in the literature, as far as the authors knowledge.

#### 3.1.2. Determination of Tocopherols

The tocopherol composition of *S. odorifera* epicarp is presented in Table 1. The sample analyzed has significant amounts of these lipophilic phytochemicals, mainly β- and δ-tocopherols (164.9 ± 0.4 and 180 ± 2 µg/100 g dw, respectively), contrary to the small amounts of α- and γ-tocopherol detected.

In the pulp fruit, about 2.35 µg of vitamin E has been quantified in 100 g of fresh mass, with α-tocopherol in the highest amounts (33.28%), followed by α-tocotrienol and δ-tocopherol (21.54% and 16.93%, respectively); and contrary to our results, γ-tocopherol has not been detected in the edible parts of *S. odorifera* [6]. Vitamin E, including tocopherols and tocotrienol, is an important phytochemical with health-promoting effects, being present in the human serum and correlated with protection against cardiovascular disorders [21]. Moreover, natural and synthetic tocopherols are used as antioxidant agents in vegetal oil products [22].

#### 3.1.3. Determination of Phenolic Compounds

The epicarp of *S. odorifera* was evaluated regarding its phenolic composition. The data obtained from HPLC-DAD-ESI/MS^n^ analysis are presented in Table 2. The tentative identification was made according to the spectrum characteristics of the detected peaks, considering their retention time (Rt), maximum absorbance wavelength (λmax), pseudomolecular ion ([M − H]^−^/[M]^+^, and the correspondent fragmentation pattern (MS^2^), and the information found in the literature were used for the confirmation of some compounds.

- Non-anthocyanin phenolic compounds

Two non-anthocyanin phenolic compounds were identified in *S. odorifera* epicarp, both belonging to the flavanols class. Peak 1 showed a pseudomolecular ion [M−H]^−^ at *m*/*z* 609 releasing a unique MS^2^ ion fragment at *m*/*z* 301 typical of aglycone quercetin. The loss of −308 u corresponds to a deoxyhexoxyl-hexoside moiety. According to its retention time, UV spectrum, and mass characteristics, this compound was tentatively identified as quercetin-*O*-deoxyhexosyl-hexoside. Peak 2, with a pseudomolecular ion [M−H]^−^ at *m*/*z* 593, showed a MS^2^ ion fragment at *m*/*z* 285, corresponding to kaempferol aglycone. Based on mass spectrum, this peak was tentative identified as kaempherol-*O*-deoxyhexosyl-hexoside.

With respect to the total non-anthocyanin phenolic compounds content, *S. odorifera* epicarp extract showed a total of 48.2 mg of flavanols/g of extract, which is equivalent to 10.1 mg/g of epicarp. The quercetin derivative was the most abundant (~78%). According to Jaramillo et al. [8], other quercetin derivatives, one kaempferol derivative, and one isorhamnetin derivative have been detected in different methanolic extract fraction, e.g., in the ethyl acetate fraction were found kaempferol 3-*O*-β-glucopyranoside and isorhamnetin 3-*O*-α-glucopyranoside, while in different fractions of the aqueous phase were identified quercetin-3-*O*-α-l-rhamnopyranosyl-(1→6)-β-d-glucopyranoside-4′-*O*-β-d-glucopyranoside, quercetin 3-*O*-β-d-galactopyranoside-4′-*O*-β-glucopyranoside, quercetin 3,4′-di-*O*-β-d-glucopyranoside, quercetin 3-*O*-(6″-O-malonyl)-β-d-glucopyranoside 4′-*O*-β-d-glucopyranoside, quercetin 3-*O-*α-l-rhamnopyranosyl-(1→6)-β-d-galactopyranoside, quercetin 3-*O*-β-l-rhamnopyranosyl-(1→6)-β-d-glucopyranoside, and quercetin-3-*O*-β-d-glucopyranoside and quercetin-3-*O*-(6″-malonyl)-βd-glucopyranoside [8]. It is important to clarify that the process of extraction, purification of the compounds, and the method of analysis employed by the authors were different from those carried out in the present study, for this reason, the phenolic profile described is not coherent (qualitatively) with other authors description. In the present study, a simple maceration extraction with ethanolic solvent was performed and the crude extract was analyzed as a whole.

- Anthocyanin compounds

Regarding anthocyanins, the sample presented also two compounds, peak 3, with a pseudomolecular ion [M]^+^ at *m*/*z* 595, depicting two MS^2^ fragment ions, one at *m*/*z* 449, after a loss of a deoxyhexosyl unit (−146 u) and another at *m*/*z* 287, which correspond to the cyanidin aglycone after a loss of an hexosyl moiety (−162 u), being tentatively identified as cyanidin-*O*-deoxyhexosyl-hexoside. Previously, Jaramillo et al. [8] detected the same pseudomolecular ion with the same fragmentation behavior in the analysis of the fractionated aqueous extract of the *S. odorifera* epicarp. According to the authors, this compound has been identified as cyanidin-3-*O*-rutinoside, however, without the application of other separation technologies is not possible, for us, to state without a doubt the exact sugar moiety and position. Peak 4 presented a pseudomolecular ion [M]^+^ at *m*/*z* 579, releasing two MS^2^ fragment ions at *m*/*z* 433 and *m*/*z* 271, the last being corresponding to the pelargonidin aglycone. As observed for peak 3, the MS^2^ fragmentation behavior of this compound also indicates the loss of a deoxyhexosyl (−146 u) and hexosyl moieties (−162 u). Thus, according to the chromatographic and MS characteristics, peak 4 was identified as pelargonidin-*O*-deoxyhexoside-hexoside. To the best of our knowledge, this is the first time that this compound was detected in this fruit. In literature, two other anthocyanins, namely, cyanidin-3-*O*-β-d-glucopyranoside and peonidin-3-*O*-(6″-*O*-*p*-coumaroyl)-β-d-glucopyranoside, have been detected in *S. odorifera* epicarp purified methanolic extract [8].

The epicarp extract showed the highest amount of anthocyanin compounds (111 ± 5 mg/g E), being cyanidin-3-*O*-rutinoside the abundant compound, corresponding to about 90% of the total anthocyanins. The total anthocyanin in dry epicarp was of 24 ± 1 mg/g dw. The concentration of this phenolic compound in *S. odorifera* epicarp fruit is interesting, since it is more than quantified in other purple fruits, such as jabuticaba (*Myrciaria jaboticaba* (Vell.) Berg) epicarp (50.1 mg/g E) [23], grape (*Vitis vinifera* L.) peel (7.9 mg/g E) [24], passion fruit (*Passiflora edulis* Sims) epicarp (9 mg/g E) [25], juçara (*Euterpe edulis* Martius) peels (11 mg/g dw) [26], eggplant (*Solanum melongena* L.) epicarp (11.9 mg/g E) [27], and açai (*Euterpe oleracea* Mart.) (100 mg/g E) [28]. This result reveals that *S. odorifera* epicarp can be a promising source of natural colorants.

### 3.2. Bioactive Proprieties

#### 3.2.1. Antioxidant Activity

The antioxidant activity of *S. odorifera* epicarp extract was evaluated thought two cell-based methods, which allowed to determine the ability of this extract to inhibit lipid peroxidation (by TBARS assay) and prevent the oxidative hemolysis (by OxHLIA assay). The results obtained are presented in Table 3. Regarding the inhibition of lipid peroxidation, the extract showed moderate antioxidant activity (EC_50_ value of 48.2 ± 0.5 µg/mL), value more than eight times higher than that obtained for Trolox control. However, *S. odorifera* epicarp extract showed higher activity than other by-products from purple fruits, such as grape skin, juçara peels, and eggplant epicarp (EC_50_ values of 629, 204, and 135 µg/mL, respectively) [24,26,27].

Regarding OxHLIA assay, a low concentration of extract (27 ± 1 µg/mL) was required to keep 50% of erythrocytes of sheep blood intact along a period of 60 min, with the extract presenting an EC_50_ value close to the one required for Trolox (21.8 ± 0.2 µg/mL), which represent a great activity for a natural extract. The anti-hemolytic activity of the extract was superior to that of other anthocyanin-rich extracts, such as passion fruit extract (EC_50_ value of 78 ± 3 µg/mL) [25], juçara peel extract (EC_50_ value of 42 ± 1 µg/mL) [26], and eggplant epicarp extract (EC_50_ value of 34 ± 1 µg/mL) [27]. These results indicate that this by-product can be explored regarding its antioxidant proprieties.

#### 3.2.2. Cytotoxicity and Hepatoxicity of the *S. odorifera* Extract

The results of the cytotoxic effects of the extract evaluated are presented in Table 3. The sample did not affect the growth of all tumor-cell lines tested at the maximum concentration tested (400 μg/mL), which supposes that its antiproliferative action against malignant cells is very low or null. On the other hand, it can be considered that the extract has no adverse effects on normal cells, since at the maximum concentration evaluated (400 µg/mL), the extract did not inhibit the growth of PLP2 cells.

Bussmann et al. [29] evaluated the toxicity of different extracts obtained from *S. odorifera* leaves using brine-shrimp assay. According to the results achieved by the authors, the aqueous extract presented a LC_50_ value (median lethal concentration) > 10,000 µg/mL, while the ethanolic extract had a LC_50_ value of 488 µg/mL. Therefore, the cytotoxicity of the plant extracts may depend on the conditions for obtaining them. In the present study, the hydroethanolic extract of the *S. odorifera* epicarp did not showed cytotoxicity. However, it is worth mentioning that no other study was found to report the toxic effect of *S. odorifera* epicarp; nevertheless, more studies are needed to determine the safe consumption parameters of the possible products derived from those by-products.

#### 3.2.3. Antibacterial Activity

*S. odorifera* epicarp extract was also evaluated regarding its antibacterial proprieties, and the results are present on Table 4. In general, the extract showed the same efficiency to inhibit the growth of all bacterial culture tested (MIC = 1.1 mg/mL) and its bactericidal effect was achieved with twice the bacteriostatic concentration (MBC = 2.2 mg/mL).

It is interesting to highlight that for some bacteria, *S. odorifera* epicarp extract was more efficient than the controls tested, such as for *B. cereus*, when higher concentration of the food additive E224 was required to inhibit growth (bacteriostatic effect), but also to kill the bacteria itself (bactericidal effect). The controls evaluated are food additive extensively applied in food industry due to their antioxidant and conservative properties [30,31], however some side effects to human health have been related in the last years, such as skin sensitization [31].

In the literature, a few studies report the antibacterial activity of *Sicana* spp. In the work carried out by Castro et al. [32], the hydroethanolic extract of *S. odorifera* epicarp did not show activity against the growth of *Lactobacillus casei* in the maximum concentration evaluated (50 mg/mL) by the authors. In other study, the ethanolic extract of *S. odorifera* leaves has shown antibacterial activity against *S. aureus* at concentration of 128 mg/mL [33], which is higher than the MIC value of the hydroethanolic extract determined for this microorganism in our study.

#### 3.2.4. Antifungal Activity

The results of antifungal activity of the *S. odorifera* epicarp extract are shown in Table 4. All fungi strains were sensitive to the hydroethanolic extracts studied, highlighting the low concentration of extract that was required to inhibit the growth of *T. viride* (MIC = 0.28 mg/mL), while higher concentration of the controls E211 and E224 (MIC = 1.0 and 0.5 mg/mL, respectively) were necessary. Additionally, *Penicillium* spp. were more susceptible to the extracts than *Aspergillum* spp. Moreover, our results show that the inhibitory action of the *S. odorifera* epicarp extract on the fungi evaluated was more effectives than the activity of some extracts obtained from other fruit by-products, such as from eggplant epicarp (MIC > 8 mg/mL for all fungi tested in this work) [27] and passion fruit epicarp (MIC values of 4.0 and 8.0 mg/mL for *A. niger* and *T. viride* and for *A. fumigatus*, *A. versicolor*, and *P. funiculosum*, respectively) [25]. However, the epicarp extract showed fungistatic and fungicidal effects, which reveals a huge potential to be explored of this by-product as an antifungal agent.

According to the literature, *S. odorifera* plant has shown high resistance against the infection of *Stagonosporopsis citrulli*, a pathogenic fungus responsible for the gummy stem blight disease in cucurbit species, phenomenon that may be correlated with the high antifungal activity of this plant [34]. Further studies are proposed to better understand the antifungal potential of this by-product regarding the inhibition of different types of fungi, since data on this particular bioactive property of *S. odorifera* fruit and its parts are scarce in the literature.

## 4. Conclusions

To the best of our knowledge, this study reported for the first time the composition in organic acids and tocopherols of *S. odorifera* epicarp, as also the antimicrobial activity of this bio-residue. *S. odorifera* epicarp also presented an interesting composition in anthocyanin and non-anthocyanin phenolic compounds. It also revealed antioxidant, antibacterial, and antifungal effects and did not show hepatotoxic effects against the non-tumor cell line PLP2. The results achieved herein show that *S. odorifera* epicarp as an interesting source of bioactive molecules for application in different segment industrial sectors, such as food and pharmaceutical industries. In addition, this work can promote the dissemination of knowledge about this fruit, thus encouraging its cultivation, commercialization, and exploitation to obtain a high added value ingredient.

## Figures and Tables

**Table 1:** Composition in organic acids and tocopherols of the *S. odorifera* epicarp.

**Organic Acids**	**g/100 g**
Oxalic acid	0.322 ± 0.003
Shikimic acid	tr
Citric acid	3.05 ± 0.08
Fumaric acid	tr
*Total*	3.38 ± 0.08
**Tocopherols**	**mg/100 g**
α-tocopherol	18.5 ± 0.7
γ-tocopherol	2.4 ± 0.2
β-tocopherol	164.9 ± 0.4
δ-tocopherol	180 ± 2
*Total*	366 ± 2
Results are presented as mean ± standard deviation. tr—traces.	

**Table 2:** Retention time (Rt), wavelengths of maximum absorption in the visible region (λmax), mass spectral data, tentative identification, and quantification estimation of compounds in *S. odorifera* epicarp.

**Peak**	Rt	λmax	[M − H]^−^/[M]^+^	MS^2^	Tentative Identification	References	Quantification	
							Extract	Epicarp
	(min.)	(nm)	(*m*/*z*)	*(m*/*z*)			(mg/g Extract)	(mg/g dw)
*Non-anthocyanins*								
1	16.5	354	609	301(100)	Quercetin-*O*-deoxyhexosyl-hexoside	DAD-MS	37.7 ± 0.2	7.91 ± 0.04
2	19.75	348	593	285(100)	Kaempherol-*O*-deoxyhexosyl-hexoside	DAD-MS	10.5 ± 0.3	2.20 ± 0.06
					TPC-non anthocyanin		48.2 ± 0.5	10.1 ± 0.1
*Anthocyanins*								
3	15.07	514	595	449(32),287(100)	Cyanidin-*O*-deoxyhexosyl-hexoside	[8]	100 ± 4	21.5 ± 0.9
4	18.54	504	579	433(21),271(100)	Pelargonidin-*O*-deoxyhexosyl-hexoside	DAD-MS	11.5 ± 0.6	2.5 ± 0.1
					TA		111 ± 5	24 ± 1
Results are present as mean ± standard deviation TPC—total phenolic compounds; TA—total anthocyanins.								

**Table 3:** Antioxidant, anti-inflammatory, and anti-proliferative activities of *S. odorifera* epicarp hydroethanolic extract.

	Epicarp Extract	Control Trolox
*Antioxidant activity (EC_50_ = μg/mL)*		
TBARS	48.2 ± 0.5	5.8 ± 0.6
OxHLIA (ΔT_60 min_)	27 ± 1	21.8 ± 0.2
*Anti-inflammatory activity (EC_50_ = μg/mL)*		Dexamethasone
RAW 264.7	>400	16 ± 1
*Anti-proliferative activity (GI_50_ = μg/mL)*		Ellipticine
NCI-H460	>400	1.0 ± 0.1
HepG2	>400	1.1 ± 0.2
MCF-7	>400	0.91 ± 0.04
HeLa	>400	1.91 ± 0.06
*Hepatotoxicity (GI_50_ = μg/mL)*		
PLP2	>400	3.2 ± 0.7
Results are presented as mean ± standard deviation.		

**Table 4:** Antibacterial and antifungal activities of *S. odorifera* epicarp hydroethanolic extract.

**Antibacterial Activity (mg/mL)**	**Epicarp Extract**		**E211**		**E224**	
	**MIC**	**MBC**	**MIC**	**MBC**	**MIC**	**MBC**
Gram-positive bacteria						
*Staphylococcus aureus*	1.1	2.2	4.0	4.0	1.0	1.0
*Bacillus cereus*	1.1	2.2	0.5	0.5	2.0	4.0
*Listeria monocytogenes*	1.1	2.2	1.0	2.0	0.5	1.0
Gram-negative bacteria						
*Escherichia. coli*	1.1	2.2	1.0	2.0	1.0	1.0
*Salmonella typhimurium*	1.1	2.2	1.0	2.0	0.5	1.0
*Enterobacter cloacae*	1.1	2.2	2.0	4.0	0.5	0.5
**Antifungal activity (mg/mL)**	**MIC**	**MFC**	**MIC**	**MFC**	**MIC**	**MFC**
*Aspergillus fumigatus*	2.2	4.4	1.0	2.0	1.0	1.0
*Aspergillus versicolor*	1.1	2.2	2.0	4.0	1.0	1.0
*Aspergillus niger*	1.1	2.2	1.0	2.0	1.0	1.0
*Penicillium funiculosum*	0.55	1.1	1.0	2.0	0.5	0.5
*Penicillium verrucosum* var. *cyclopium*	0.55	1.1	2.0	4.0	1.0	1.0
*Trichoderma viride*	0.28	0.55	1.0	2.0	0.5	0.5

## Data Availability

Data is contained within the article.
